# Cluster analysis of medical students’ attitudes regarding people who use drugs: a first step to design a tailored education program

**DOI:** 10.1186/s12909-024-05380-8

**Published:** 2024-05-03

**Authors:** Lou Richelle, Michele Dramaix-Wilmet, Quentin Vanderhofstadt, Charles Kornreich

**Affiliations:** 1 Unité de Recherche en Soins Primaires ULB, Faculty of Medicine, Université Libre de Bruxelles, Brussels, Belgium; 2 Département de Médecine Générale, Faculty of Medicine, Université Libre de Bruxelles, Brussels, Belgium; 3 Département d’Epidémiologie et de Biostatistiques, School of Public Health, Université Libre de Bruxelles, Brussels, Belgium; 4 Laboratoire de Psychologie Médicale et d’Addictologie, Faculty of Medicine, Université Libre de Bruxelles, Brussels, Belgium

**Keywords:** Substance use disorder, Attitudes, Medical students, Cluster analysis, Education

## Abstract

**Introduction:**

People with substance use disorder (SUD) deal with stigmatization in various areas of life, including healthcare system. In this study, we investigated the attitudes of final-year medical students towards SUD people and attempted to understand their influence.

**Methods:**

We conducted a two-stage cluster analysis (hierarchical ascending classification followed by K-means clustering) based on the “beSAAS”. We administrated this 23-item questionnaire to 923 final-year medical students in Belgium (response rate = 71,1%). Sociodemographic characteristics were compared between the clusters.

**Results:**

Four clusters of students with specific characteristics were identified in this study. The first, “The Inclusives” (including 27,9% of respondents) had the least negative attitudes; they wanted to specialize mainly in psychiatry and gynecology. The second, “The Centrists" (23,6%) consisted mainly of male students. They had many private and professional experiences with substance use and considered themselves less healthy than others did. Most wanted to specialize in pediatrics and general practice. Their attitudes were slightly negative towards people with SUD. The third, “The Moralists” (27,6%), were mainly older, from non-European countries, had the least experience with substance use (or contact mainly in hospitals), had the less high mother’s level of education and reported excellent health. They were heading toward other specialties. They had the most stereotypes and moralism, and less treatment optimism. The fourth, “The Specialist care-oriented” (20,8%), were the most in favor of specialized treatment. This group had a higher proportion of Belgian, females, and students who had specific contact with this population. They especially intended to specialize in internal medicine.

**Conclusion:**

This study revealed 4 profiles of medical students with different attitudes towards SUD people. “The Moralists”, including more than a quarter of the respondents, were characterized by strong stereotypes and moralism and little treatment optimism. These clusters could contribute to the design of a learner-centered program aimed at addressing stigma within the main curriculum.

**Supplementary Information:**

The online version contains supplementary material available at 10.1186/s12909-024-05380-8.

## Introduction

Substance use disorder (SUD) is a challenging public health issue affecting 39,5 million people worldwide, according to the World Drug Report 2023 [1]. Room et al. (2001) highlighted that among the 18 most stigmatized social problems in 14 countries, drug addiction was ranked number one, and alcohol addiction was ranked number four [2]. The stigma process could be understood as “*a process in which people are firstly labeled and thereby assigned to an out-group, secondly subjected to stereotypes and prejudices, and thirdly exposed to discrimination and social distance. This dynamic process implies a nonlinear and interactive stigma process, in which each component can mutually reinforce the others*” [3]. People who use drugs (PWUD), particularly illicit substances, face numerous stigmas, including health professionals [4–7]. Stereotypes and prejudices are common and may lead to substandard care by health professionals and limited access to the healthcare system [6–9]. Health professionals may show less engagement and adopt a more task-oriented approach to healthcare delivery, which can lead to diminished empathy [7]. Negative attitudes can reduce patients' sense of empowerment and recovery optimism and negatively impact treatment outcomes (unmet needs, postponing treatment, discontinuing treatment, etc.) [6–8, 10].

The literature reports several factors impacting stigmatization by health professionals such as age, gender, personal and professional experiences with SUD [5, 7, 9, 11–13]. Medical students and doctors are also concerned by stigma, which is more pronounced in certain disciplines [9, 12–15].

In this study, we aimed to assess the attitudes of medical students about PWUD and to determine whether there were profiles of students who shared certain characteristics in terms of attitudes as well as to understand what could explain them. Our aim was to adapt the addiction training on this basis. We chose the beSAAS questionnaire (see Table S1 Appendix A) [16] to survey final-year medical students. We focused on this population, as they are future generations of doctors.

## Materials and methods

### beSAAS questionnaire

The beSAAS Questionnaire is a 23-item questionnaire. It is the result of an exploratory factor analysis we have performed in a previous study on the bSAAS, a French adapted version of Substance Abuse Attitude Survey (SAAS), an internationally validated questionnaire which we had chosen because it best suited our needs [16]. The exploratory factor analysis retained twenty-three items correlated to three factors; namely, “Stereotypes and moralism”, “Treatment optimism” and “Specialized treatment” with 70% of total variance explained and Cronbach’s alpha = 0.80 The first factor called “Stereotypes and moralism” includes items such as “Pregnant women who use drugs should be punished”. The second factor “Treatment optimism”, contains items such as “Drug addiction is a treatable illness”. And the last factor “Specialized treatment”, with items such as “Alcohol addiction should only be treated by specialists in that field”. Each item was coded according to a Likert scale ranging from 1 (strongly disagree) to 5 (strongly agree). To be noted that the score “Stereotypes and moralism” was positively correlated with the “Specialized treatment” score and negatively correlated with the “Treatment optimism” score (the correlations were weak to moderate).

In order to characterize our population and to better understand the factors influencing the students’ representations, we included several data sources: socio-demographic data (gender, age, origin), data related to the personal and professional background linked to substance use, data related to the type of professional orientation (choice of speciality), as well as data related to the perception of their own health (we wanted to evaluate whether this influenced the way in which people with SUD are perceived).

### Data collection

The questionnaire was presented to 923 final-year medical students (Université Libre de Bruxelles) of three consecutive years (2019, 2020 and 2021). The final-year corresponds to the 6th year of the main medical curriculum in Belgium. It was administered face-to-face to students in 2019 and online in 2020 and 2021 given the context of the SARS-CoV-2 crisis. A total of 657 students completed the questionnaire with an average response rate of 71.1%. Eighty students completed the questionnaire at the time of enrollment in a voluntary theoretical addictology course offered by the Department of General Practice. Records with missing items were not considered. One or two items were missing in thirty-two (4.9%) of the questionnaires and three to eleven items in ten (1.5%) of questionnaires. On this basis, 615 (93.6%) questionnaires were retained.

### Statistical methodology

Cluster analysis was performed based on the beSAAS Questionnaire (including the 23 items and the 3 factor scores derived from the exploratory factor analysis). A hierarchical ascending classification based on the WARD method was first used (Calinski/Harabasz and Duda/Hart criteria), we decided to keep 4 clusters. K-means clustering was then applied to the result obtained by the ascending hierarchical classification [17]. The mean factor scores were compared between the 4 clusters using one-way analysis of variance (ANOVA), followed by 2-by-2 multiple comparisons with Bonferroni correction. The distribution of subjects’ characteristics were compared between the 4 clusters using the Chi^2^ test. To analyse the relationship between the clusters and some items of particular interest of questionnaire, we used correspondence analysis. The analyses were performed with STATA SE V16.1 software for all analyses and the significance level was set at 5%.

## Results

### Clusters characteristics

Cluster analysis allowed us to identify four clusters. The first cluster, which we called "The Inclusives", corresponded to 27.9% of the respondents. It was characterized by the lowest average scores on the "Stereotypes and Moralism" factor and the "Specialized treatment" factor. The respondents included in this cluster scored the highest for "Treatment optimism”. The second cluster, which we called "The Centrists," consisted of 23.6% of respondents whose means in the three factors were closest to the overall mean in all factors. They were not particularly positioned in favor of any factor, scoring slightly higher on the “Stereotypes and moralism" factor and slightly lower on the "Treatment optimism" and "Specialized treatment" factors. The third cluster, which represented 27.6% of respondents, was called "The Moralists" because they scored the highest on "Stereotypes and moralism”. Respondents were also in favor of “Specialized treatment” and were not very optimistic about treatment. The final cluster, consisting of the smallest cluster (20.8% of respondents), was called "The Specialist care-oriented" because they scored the highest for this factor. Otherwise, they scored low on "Stereotypes and moralism" and scored slightly above average for “Treatment optimism”. See Table 1.

**Table 1:** Clusters and factorial scores

Factorial scores	Cluster 1	Cluster 2	Cluster 3	Cluster 4	Total
	Inclusives *n* = 172	Centrists *n* = 145	Moralists *n* = 170	Specialist care-oriented *n* = 128	*n* = 615
	Mean (SD)	Mean (SD)	Mean (SD)	Mean (SD)	Mean (SD)
Stereotypes & moralism	1.56 (0.32)	2.23 (0.39)	2.61 (0.40)	1.85 (0.31)	2.07 (0.55)
Treatment Optimism	3.19 (0.36)	2.84 (0.36)	2.69 (0.35)	3.01 (0.33)	2.94 (0.40)
Specialized treatment	1.95 (0.44)	2.16 (0.28)	2.94 (0.45)	3.26 (0.41)	2.55 (0.67)

One-way ANOVA: *p* < 0.001 for the 3 scores. After Bonferroni correction: significant differences (*p* ≤ 0.001) between each pair of clusters

**Fig. 1:**
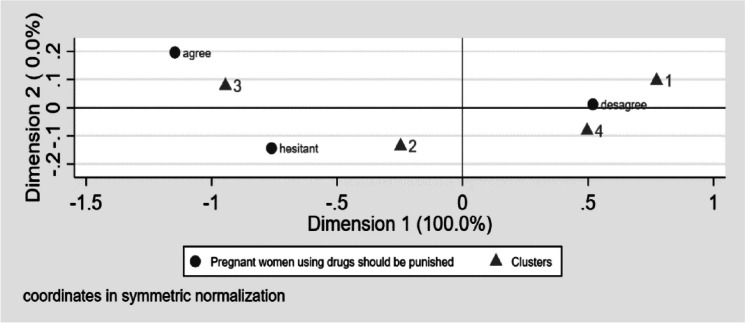
Clusters attitudes regarding to the item about punishing substance use during pregnancy Cluster 1 = The Inclusives, Cluster 2 = The Centrists, Cluster 3 = The Moralists and Cluster 4 = The Specialist care-oriented

To confirm our model, we looked at some items that were particularly determinant of the factors and analyzed the positions of the clusters with regard to those items based on correspondence analyses (See Fig. 1). For instance we used the item about punishing pregnant women using drugs, the most weighted in the factor “Stereotype and moralism”. The results were consistent and confirmed our choice of clusters’ names. Indeed, we can observe that “The Moralists” (Cluster 3) agreed with punishing substance use in pregnant women, “The Centrists” (Cluster 2) were not positioned, “The Specialist care-oriented” (Cluster 4) disagreed and “The Inclusives” (Cluster 1) strongly disagree.

We also highlighted some differences in attitudes toward alcohol and drugs. Noteworthy distinctions include a slightly higher agreement on perceived low willpower linked to drug use compared to alcohol (15,3% vs 13%), especially present among “The Centrists” and “The Moralists”. Respondents in favor of punishing pregnant women consumers leaned slightly more towards alcohol than drugs (16,8% vs 13,2%). This trend was particularly pronounced among “The Moralists” (34,7% vs 27,6%). Respondents expressed a slight preference for specialized treatment in alcohol-related issues compared with drug-related issues, which was notably pronounced among “The Specialist care-oriented” (20,3% vs 13,3%). Optimism about treatment outcomes remained consistent between the two substances. All the associations were statistically significant.

Sociodemographic characteristics of respondents according to clusters.

65,11% of the respondents were female. “The Centrists” included the highest proportion of male respondents (39,4%), hardly more than”The Moralists” (38,8%). At the opposite, “The Specialist care-oriented” were characterized by the highest number of female gender students (74,60%).

As shown in Table 2, “The Inclusives" had the lowest level of older students (more than 30 years old) and “The Centrists" were marked by the highest level of mother’s education and lower health perception (satisfactory to good). Regarding “The Moralists”, they were characterized by the oldest students, the fewest students with a high mother’s level of education and the best perception of their health. Finally, “The Specialist care-oriented” stand out except for the most “middle” maternal education level.

**Table 2:** Subjects’ socio-demographic characteristics in regard of the clusters

Subjects’ socio-demographic characteristics	Cluster 1	Cluster 2	Cluster 3	Cluster 4	Total
	Inclusives *n* (%)	Centrists *n* (%)	Moralists *n* (%)	Specialist care-oriented *n* (%)	*n* = 615
	172 (27,9)	145 (23,6)	70 (27,6)	128 (20,8)	
**Gender**					
Female	65,7	60,56	61,21	74,60	65,1
Male	34,3	39,4	38,8	25,40	34,9
**Age (years)**					
< 25	55.4	58.5	48.2	58.4	54.8
25–29	42.2	37.3	44.5	36.8	40.5
> 30	2.4	4.2	7.3	4.8	4.7
*p* = 0.25					
**Maternal education level**					
Low	8.6	5.1	9.5	9.8	8.3
Medium	19.1	19.0	22.2	14.8	19.0
High	72.2	75.9	68.4	75.4	72.7
**Health perception**					
Excellent	26.3	24.7	30.8	28.6	27.7
Very good	49.2	44.7	49.5	46.2	47.6
Good-satisfactory	24.6	30.6	19.6	25.3	24.7
*p* = 0.75					

With regard to origin, we observe that “The Inclusives” group included the most European students (22,1%); “The Centrists” the most Belgian students of European origin (23,2%); “The Moralists” the most students of non-European origin (17,9%) and “The Specialist care-oriented” the most of Belgian students (53,6%), (*p* = 0,001). See Figure S1 Appendix B.

In terms of substance experiences, “The Inclusives” stood out by the highest level of personal consumption of cannabis and the most other substance disorders in the entourage. “The Centrists” by the most personal substance use (cannabis and multiple) and SUD in the entourage (notably alcohol-cannabis use disorders). “The Moralists” by the less personal use or SUD in the entourage. And “The Specialist care-oriented” by the highest level of cannabis use disorder in the entourage. The details are presented in Table 3.

**Table 3:** Substances experiences in regard of the clusters

Substances experience	Cluster 1	Cluster 2	Cluster 3	Cluster 4	Total
	Inclusives *n* = 172	Centrists *n* = 145	Moralists *n* = 170	Specialist care-oriented *n* = 128	*n* = 615 (100)
	%	%	%	%	%
**Drugs consumption**					
No	52.8	50.0	63.0	56.8	55.8
Cannabis	37.4	36.4	24.2	30.4	32.0
Multiple	9.8	13.6	12.7	12.8	12.1
*P* = 0.16					
**SUD in the entourage**					
No	37.4	36.0	39.6	37.7	37.8
Alcohol	15.3	12.2	16.5	11.5	14.1
Cannabis	4.3	6.5	6.7	11.5	7.0
Alcohol-cannabis	16.0	19.4	15.2	13.9	16.2
Other drugs	27.0	25.9	22.0	25.4	25.0
*P* = 0.65					

Regarding the professional trajectory, “The Inclusives" had the most contacts in general practice (GP) followed by many specific contacts (i.e., addiction centers, prisons, psychiatry clinics, etc.). They wanted most to specialize in gynecology, psychiatry (adult and child), and GP in the second position. They were also more willing to participate in addictology training (offered by the GP Department). “The Centrists” had had the most contacts in multiple contexts. They wanted to specialize in pediatrics and GP with internal medicine in second place. “The Moralists” had the least contact with patients with SUD or especially in a hospital setting. They were most interested in going into other specialties, including surgery, anesthesia, and emergency, and registered the least with optional addiction training. “The Specialist care-oriented” had the most contact in specific settings and the least in GP. They wanted to specialize the most in internal medicine. See Table 4.

**Table 4:** Subjects’ professional trajectory

Subjects’Professional career	Cluster 1	Cluster 2	Cluster 3	Cluster 4	Total
	Inclusives *n* = 172	Centrists *n* = 145	Moralists *n* = 170	Specialist care-oriented *n* = 128	*n* = 615 (100)
	%	%	%	%	%
**Contacts with SUD people**					
Specific contacts	10.9	5.7	5.5	11.1	8.2
Multiple	50.9	56.7	44.2	50.0	50.3
General practice	4.9	3.6	4.2	1.6	3.7
Hospital	30.9	30.5	40.6	33.3	34.0
No	2.4	3.6	5.5	4.0	3.9
*p* = 0.23					
**Choice of medical speciality**					
General practice	26.7	31.7	21.8	23.4	25.9
Internal medicine	12.8	13.8	10.6	14.1	12.7
Paediatrics	5.2	8.3	7.1	6.3	6.7
Gynaecology	9.3	3.5	6.5	7.0	6.7
Psychiatry	5.2	2.1	0.6	3.1	
Other	45.9	42.8	54.1	49.2	48.1
*p* = 0.46					
**Optional addiction training**					
No	86,6	89,7	94,1	89,1	89,2
Yes	13,4	10,3	5,9	10,9	10,1
*p* = 0,14					

**Fig. 2:**
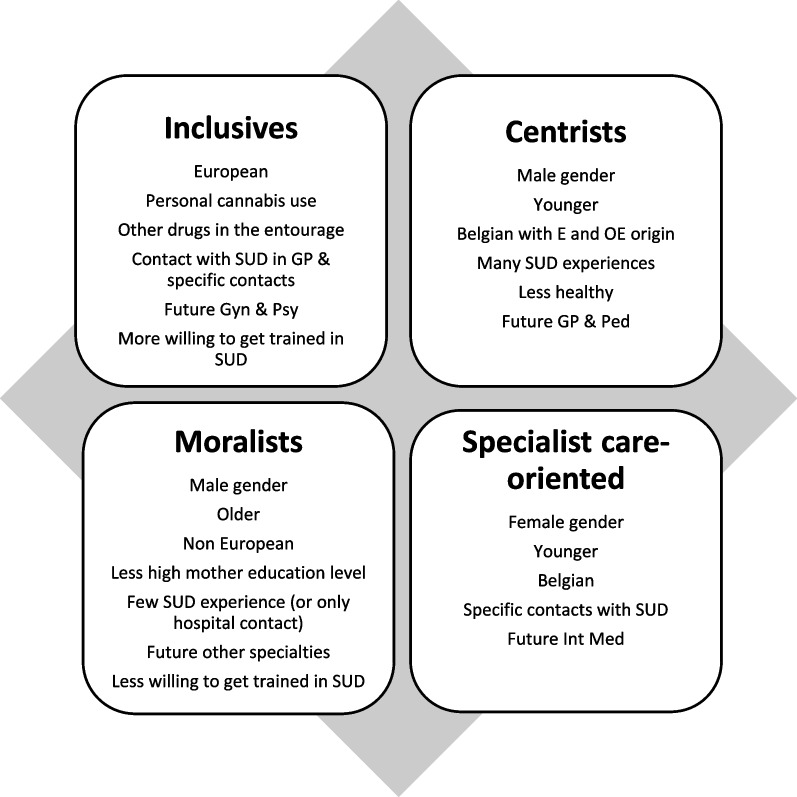
Main characteristics of the four clusters GP = general practice, Gyn = gynaecology, Int Med = internal medicine, OE = outside Europe, Psy = psychiatry (adult and child), Ped = paediatric, SUD = Substance use disorder

We synthetized all the main characteristics of each cluster in Fig. 2.

Additionally, to investigate if the SARS-CoV-2 crisis could have impacted students’ representations, we compared factors and variables according to the three student cohorts (2019, 2020 and 2021). The results did not show any relevant significant difference except for a slight decrease in the average score of Factor I (Stereotypes and moralism) over the years (from 2.14 to 2.02; *p* = 0.05)^20^.

## Discussion

Our results are challenging and provide a better understanding of the attitudes of future doctors and their influences. The fact that this study revealed four distinct groups in terms of attitudes towards SUD people with specific socio-demographic characteristics, life, and professional experiences with substances is an important contribution of the study and allows us to identify potential actions to address stigma. We did not find any similar research in literature.

Concerning these influencing factors, this study explored new items of interest. Indeed, if the questions of age, gender, and experience related to substance use among medical students and doctors had already been studied in previous studies as mentioned above, this study allowed us to compare medical students on different aspects not yet studied, such as their choice of orientation, which, to our knowledge, has never been explored before, especially among medical students. This makes it possible to compare the attitudes of all profiles of future specialities and not only a few of them, as highlighted previously in the literature [9, 12]. Another characteristic that seems to have never been studied is the perception of their own health in relation to their representations of PWUD.

Regarding the four clusters, several items were interesting to discuss. For “The Inclusives", it is interesting to note that contact with people with SUD in GP or in specific centers had globally a positive impact on their attitudes as they had the most contact with SUD in these settings. A limited literature showed indeed less stigma in primary care [12, 14]. The majority of these students wanted to specialize in gynecology, psychiatry, and GP (cluster with the second highest percentage). All three specialities are particularly interested in the psychosocial and biological dimensions of human beings. These students are undoubtedly particularly sensitive to a person-centered and empathetic approach. The literature is mixed on the association between empathy and career choice, but it tends to show more empathy in students who go into a people-oriented specialty such as GP or psychiatry and a more technical orientation such as surgery [18, 19]. This choice may also be explained based on personality traits [20].

"The Centrists" is a cluster marked by a certain level of knowledge about substances, in the students' personal and professional narrative. These students had the most multiple contacts with PWUD but the least specific contacts, which could partly explain their attitudes. Indeed, contact with this population in settings where they are not well received or treated could also impact them as they may integrate the negative attitudes of their mentors [15, 16]. Otherwise, we assumed before conducting this study that feeling less healthy, potentially having chronic health problems or mental health disorders, meaning being more vulnerable, could have a positive impact on their sensitivity to empathy and others’ vulnerability but surprisingly this did not seem to be the case. SUD being commonly not seen as a chronic disease, even by the health professionals [5–7]. However, perhaps because in this cluster, respondents had many disorders related to cannabis and alcohol use in their entourage. Both are trivialized and are complex conditions with no efficient medication. As future doctors, members of this cluster may have ambivalent relationships with this condition.

The “Moralists” correspond to the characteristics of students who were in favor of punishing pregnant women using substance in our previous study [21]. Members of this cluster were male and older students whose mothers had lower education levels or no personal or SUD in their entourage. They also had limited contact with people with SUD (i.e., none or limited to the hospital). This is confirmed in Fig. 1. The original component was identified in the present study. Indeed, the majority of students in this cluster came from non-European countries. Finding more male students in this cluster is in line with the literature on mental illness and SUD stigma in health professions students [21, 22]. That could be explained by the fact that female healthcare workers are known to be more empathetic [18, 19, 21]. Attitudes according to gender are controversial in the general population [23, 24].

Studies showed that being familiar with substances usually lower SUD stigmas [5, 7, 21, 24]. The fact that the respondents in “The Moralists” cluster had very little experience, especially in the hospital environment, explained that they either constructed their knowledge and attitudes on the societal representation of PWUD or by their experience in the hospital environment where structural stigma can be particularly strong. It appears from the meta-analysis of stigma in mental health conducted by Petkani et al. in 2018 that clerkship may have a robust effect in combating stigma, but only if it is done in a supportive setting (e.g., psychiatry, addiction centers, etc.) [22]. This cluster included numerous future surgeons, anesthetists, and emergency physicians, which is in line with the limited literature [9, 25–27] that highlights strong stigma in these specialities, but with almost no comparison to others. Here, it is interesting to observe the impact of role modelling on these students. Role modelling is understood as: *"a teaching by example and influencing students in an unintentional, unaware, informal and episodic manner*" [28]. Indeed, as we have already highlighted in our previous study [16], it seems that students in search of an identification model at the start of their career choice already endorsed the negative attitudes and stigmas particularly conveyed in these specialities. We can therefore see the impact of the informal and hidden curriculum on those attitudes [16, 18, 28].

Concerning the origin, we could find among the “Moralists” a majority of people coming from Nord Africa (especially Morocco and Algeria) followed by students from DRC and Cameroon in this cluster consisting mainly in non-European students, which it is in line with the Belgium immigration trends [29]. The impact of dominant religions in these countries, such as Islam and Christianism, could influence their representations. Concerning Christianity, it is important to note its particularity in African countries, represented mainly by Pentecostalism and Evangelicalism. The relationship with substance use disorder tends to be particularly moralistic [30, 31]. In the literature about Islam views, this question is more nuanced and it depends on Imams’ SUD conceptions [31, 32]. This is only a hypothesis to try to understand these attitudes, which needs to be taken carefully. Observing older students in this cluster can be explained by the fact that empathy may tend to lower with time and negative representations of crystallize [5, 18, 21], but also because students coming from other countries have often had an academic and professional background before arriving in Belgium. Since they have limited personal or family consumption, they may have forged their representations according to the societal representations of their countries of origin and their professional experiences. SUD being commonly not seen as a chronic disease but as a moral failure and a lack of will, even by the health professionals [5–7]. We find no equivalent in the literature to the tendency that students from a less educated environment are more judgmental of people with SUD than those from a higher educated environment. One study highlights less stigma among people with low incomes compared to opiate addiction, which they explain by greater familiarity with opiates in this setting [24].

To end, the fact that people who consider themselves to be in excellent health are the most judgmental reinforces our initial hypothesis that it could be more difficult for some to put oneself in the place of the other person, and therefore to be empathetic with this condition when you are not suffering from anything yourself..

Finally, concerning cluster 4, “The Specialist care-oriented”, it is interesting to note that they had had the most contact with PWUD in specific SUD settings with this population but by far the least contact in GP. We can therefore assume that their attitudes are not only based on their experience, but also on the common belief explained in our previous study that complex situations must be treated by specialists [16]. The curriculum in Belgium is also very hospital-centered, not very oriented towards general practice, which has however proven to play a key role in this issue [33, 34]. Their preference for specialized treatment for alcohol over drugs can be attributed to their greater exposure to alcohol-related issues during their medical internships, primarily conducted in hospital setting [21]. Alcohol is also a significant part of our culture in Belgium, where consumption is higher than the European average, already the highest globally. This extends to university traditions, forming a familiar aspect for students from Belgium or Europe [35].

### Potential actions

In terms of perspectives, we should implement a more comprehensive and mandatory addiction training including theory, contact with people in recovery and supervised practices in appropriate settings for all medical students on a regular basis [15, 16, 22, 36]. Our study data could therefore help to shape a more learner-centred and effective training [37, 38]. Indeed, we could get inspired by the four clusters as a basis to design “Learner Personas”. “*A learner persona is the narrative about a fictionalized learner based on some common characteristics of a type of learners, such as their needs, goals, values, and attitudes in the learning contexts to be designed for them”* [37].

We should also train students more on vulnerability and empathy during medical studies, hard skills are still valued and considered more than soft skills in medical professions training. By working on SUD and stigma education we hope to slowly influence the powerful impact of informal and hidden curricula (via role modelling and clinical experiences) on medical students’ representations [39, 40].

In addition, we were able to identify certain limitations to the study. Indeed it is monocentric, the sample size was limited and its representativeness was impacted by the Sars-Cov-2 crisis (response rate fell to 47.3% in the first year of the crisis). Since it was a study by questionnaire, we were not able to evaluate the representations and attitudes of the students in real-life situations. We can hypothesize that there is a certain bias of social desirability that tends to make the students answer what they imagine to be expected or to an ideal that they make of the answers but that in reality is impacted by a series of human and contextual factors. The internal validity of the clusters seems to be acceptable (thanks to the Calinski index which is a good marker of internal validity) [17, 41]. However, we do not have the possibility to assess its external validity since we have not found any comparable study so far. It would also be interesting to be able to test the study’s reliability (especially its reproducibility).

As perspectives, it would be relevant to conduct a multi-centric study to consolidate our statistical model in order to compare it to physicians and other health professionals. In the future, the link between personality traits, choice of specialty, substance use, and substance-related representation could be further explored. As mentioned above, we could also use the results of this study to conduct further studies in order to design “Learner Personas “ to better answer training needs in addiction [37].

## Conclusion

This study shows that stereotypes of moralism are very present among final year medical students in in Belgium. Cluster analysis revealed four different clusters of respondents: "The Inclusive", "The Centrists", "The Moralists" and "The Specialist care-oriented" with regard to attitudes toward SUD. This study shows that there were influenced by certain socio-demographic characteristics and by their personal and professional experiences with substances. “The Moralists", who represented more than a quarter of the sample (27.6%), were marked by very stereotypical and moralistic attitudes. Considering this population as future caregivers, it is necessary to work on addressing SUD stigmas in the curriculum. The use of cluster analysis, therefore, could serve as a foundational framework for the development of a learner-centered program.

### Supplementary Information

**Supplementary Material 1.**

## Data Availability

The dataset used and/or analysed during the current study are available from the corresponding author on reasonable request.
