# Correction: Cluster analysis of medical students’ attitudes regarding people who use drugs: a first step to design a tailored education program

**DOI:** 10.1186/s12909-024-05724-4

**Published:** 2024-07-19

**Authors:** Lou Richelle, Michele Dramaix-Wilmet, Quentin Vanderhofstadt, Charles Kornreich

**Affiliations:** 1 Unité de Recherche en Soins Primaires ULB, Faculty of Medicine, Université Libre de Bruxelles, Brussels, Belgium; 2 Département de Médecine Générale, Faculty of Medicine, Université Libre de Bruxelles, Brussels, Belgium; 3 Départe- ment d’Epidémiologie et de Biostatistiques, School of Public Health, Université Libre de Bruxelles, Brussels, Belgium; 4 Laboratoire de Psychologie Médicale et d’Addictologie, Faculty of Medicine, Université Libre de Bruxelles, Brussels, Belgium

**Correction: Richelle et al. BMC Medical Education (2024) 24:490**

10.1186/s12909-024-05380-8

Following publication of the original article [1], the authors identified some spelling mistakes in the Tables 1, 2 and 3 and in the Supplementary material.

The original article has been corrected.
